# Association of Apoptosis-Mediated CD4^+^ T Lymphopenia With Poor Outcome After Type A Aortic Dissection Surgery

**DOI:** 10.3389/fcvm.2021.747467

**Published:** 2021-11-12

**Authors:** Wei Luo, Jing-Jing Sun, Hao Tang, Di Fu, Zhan-Lan Hu, Hai-Yang Zhou, Wan-Jun Luo, Jun-Mei Xu, Hui Li, Ru-Ping Dai

**Affiliations:** ^1^Department of Anesthesiology, The Second XiangYa Hospital, Central South University, Changsha, China; ^2^Department of Cardiovascular Surgery, The Second XiangYa Hospital, Central South University, Changsha, China; ^3^Department of Anesthesiology, XiangYa Hospital, Central South University, Changsha, China; ^4^Department of Cardiovascular Surgery, XiangYa Hospital, Central South University, Changsha, China

**Keywords:** CD4^+^ T lymphopenia, apoptosis, major adverse events, outcomes, aortic dissection

## Abstract

**Background:** Many patients with type A aortic dissection (AAD) show low lymphocyte counts pre-operatively. The present study investigated the prognostic values of lymphopenia and lymphocyte subsets for the postoperative major adverse events (MAEs) in AAD patients undergoing surgery, and explore mechanisms of lymphopenia.

**Methods:** We retrospectively analyzed pre-operative lymphocyte counts in 295 AAD patients treated at two hospitals, and evaluated their correlation with MAEs. We prospectively recruited 40 AAD patients and 20 sex- and age-matched healthy donors (HDs), and evaluated lymphocyte subsets, apoptosis, and pyroptosis by flow cytometry.

**Results:** Multivariable regression analysis of the retrospective cohort revealed pre-operative lymphopenia as a strong predictor of MAEs (odds ratio, 4.152; 95% CI, 2.434–7.081; *p* < 0.001). In the prospective cohort, lymphocyte depletion in the AAD group was mainly due to loss of CD4^+^ and CD8^+^ T cells as compared with HDs (CD4^+^ T cells: 346.7 ± 183.6 vs. 659.0 ± 214.6 cells/μl, *p* < 0.0001; CD8^+^ T cells: 219.5 ± 178.4 vs. 354.4 ± 121.8 cells/μl, *p* = 0.0036). The apoptosis rates of CD4^+^ and CD8^+^ T cells were significantly higher in AAD patients relative to HDs (both *p* < 0.0001). Furthermore, the pre-operative CD4^+^ T cells count at a cut-off value of 357.96 cells/μl was an effective and reliable predictor of MAEs (area under ROC curve = 0.817; 95% CI, 0.684-0.950; sensitivity, 74%; specificity, 81%; *p* < 0.005). Pre-operative lymphopenia, mainly due to CD4^+^ T cells exhaustion by apoptosis, correlates with poor prognosis in AAD patients undergoing surgery.

**Conclusion:** Pre-operative lymphopenia in particular CD4^+^ T lymphopenia via apoptosis correlates with poor prognosis in AAD patients undergoing surgery.

## Introduction

Stanford type A aortic dissection (AAD) is a life-threatening cardiovascular emergency with a high risk of death if not swiftly corrected through surgery (1). Despite recent advances in surgery and organ protection, surgical intervention is associated with high mortality and morbidity (2). However, the mechanisms underlying surgical recovery remain unclear. Thus, better understanding of the mechanisms driving prognosis may help develop novel therapeutic strategies for peri-operative multiple organ protection during AAD surgery.

Mounting evidence indicates that immunomodulation of lymphocytes plays a critical role at wounds or damage at other organs (3, 4). Lymphocyte counts have been widely used as markers of systemic immune changes. Studies have revealed association between preoperative lymphopenia and heightened risk of infection after liver transplantation surgery (5), as well as myocardial injury in patients undergoing non-cardiac surgery (6). Low lymphocyte counts independently correlate with mortality and urgent need for transplantation following heart failure (7). A recent study found reduced T-cell levels and elevated B-cell counts in AAD patients (8), but the extent to which lymphocytes are involved in the prognosis after AAD surgery is not known. Particularly, low CD4 cell count has been demonstrated to be a potent marker of excessive immunosuppression in sepsis and renal transplant recipients (9, 10). Severe or transient lymphopenia in sepsis is well-known to inhibit T cell immunity (11). However, it is unknown the incidence of lymphopenia and its association with postoperative outcomes in the AAD patients undergoing aorta arch surgery, a complex surgery with cardiopulmonary bypass (CPB) and various postoperative complications.

In this study, we investigated the impact of preoperative lymphopenia on postoperative AAD outcomes. We retrospectively analyzed the association between lymphocyte count and postoperative major adverse events (MAEs). Additionally, we prospectively studied the lymphocyte subsets involved in the prognosis of postoperative adverse events, and to elucidate the potential mechanisms underlying lymphopenia in AAD patients.

## Materials and Methods

### Retrospective Clinical Data Collection and Analysis (Cohort 1)

We retrospectively identified all Stanford type-A aortic dissection patients undergoing surgery between April 2017 and April 2019 at Xiangya Hospital and the Second Xiangya Hospital of Central South University, China. The dissection is considered as AAD based on the onset of symptoms <14 days prior to admission (12). Inclusion criteria: All Stanford type-A aortic dissection patients undergoing surgery. A total of 317 patients with AAD were identified. Exclusion criteria: Patients were excluded because of pregnancy, as were those with infection, immunodeficiency syndrome, cancer, sub-acute or chronic dissection and those with missing data of preoperative blood cell count. The remaining 295 patients were included in the study population (Supplementary Figure 1).

### Prospective Lymphocyte Subset Characterization Data (Cohort 2)

A cohort of 40 consecutive AAD patients receiving total arch replacement were prospectively identified and recruited at the time of admission between June 2019 and January 2020. Inclusion criteria: All Stanford type-A aortic dissection patients undergoing surgery. Exclusion criteria: The study subjects with immunodeficiency syndrome, cancer, sub-acute or chronic dissection, coronary heart disease, diabetes, heart failure and cerebral vascular disease were excluded. And those who recently had a surgery or infectious diseases were also excluded (Supplementary Figure 2). Fresh blood samples for all experiments were collected within an hour before the induction of anesthesia. A control group consisted of 20 healthy age- and gender-matched subjects, each providing a single morning blood sample. Only healthy control subjects were recruited (Supplementary Table 1). All prospective participants provided written informed consent. Ethical approval for the study was granted by the institutional medical ethics review board of the Second Xiangya Hospital. This study was registered in Chinese Clinical Trial Registry (ChiCTR), with registration number: ChiCTR1900023815.

All of the methods were in accordance with the Declaration of Helsinki. All baseline data were done by one member in research team, and outcome documents were collected by a different team member blinded to baseline data. All documentation was analyzed by a third member in group.

### Surgical Procedure

Peri-operative surgical management and clinical practices at the two centers were similar and followed the procedure previously described (13–15). In brief, arterial cannulation was done through the right axillary artery. Femoral artery cannulation was occasionally chosen in the case of dissection in the right axillary artery or high pump pressure. Antegrade cerebral perfusion was started after the arrival of target cooling temperature. After completing the anastomosis, perfusion in the lower body was resumed, the CPB flow was gradually returned to 2.0–2.4 L/m^2^/min, and rewarming was initiated. During the rewarming phase, the branches of the aortic arch were reconstructed. After operation, the patients were transported to the intensive care unit (ICU).

### End Points

The primary end point was the incidence of MAEs during hospitalization. Postoperative complications included acute kidney injury (AKI), infection, arrhythmia, myocardial infarction, cerebrovascular accident, spinal cord injury, re-intubation, re-operation. Mortality was defined as in-hospital mortality. Patients meeting at least one criterion, were classified as suffering from postoperative MAEs.

### Data Collection and Definitions

The database included pre-operative demographic data, medical history, laboratory results, intraoperative surgical related factors and postoperative complications. Malperfusion was defined as occlusion of the vessels observed by contrast-enhanced CT or the symptom of ischemia or infarction. Arrhythmia is defined as any clinically apparent heart rhythm disturbance, including atrial fibrillation, supraventricular tachycardia, and sudden cardiac arrest. Infection includes one or more of the following: pneumonia, deep sternal wound infection, urinary tract infection, and septicemia. AKI is defined as a two-fold increase in baseline creatinine, or the need for renal replacement therapy (16). Cerebrovascular accident include transient ischaemic attack, stroke, or cerebral haemorrhagic events during postoperative period (17). Mechanical ventilation time is defined as the period between patient admission into ICU and extubation. Re-intubation was defined as re-intubation for any reason during hospitalization after extubation. Re-operation was defined as the need for re-operation for any reason during hospitalization after the initial cardiac procedure (18).

### Leukocyte Quantification

Leukocyte analysis was done on blood collected in ethylene diaminetetra acetic acid (EDTA)-treated tubes using automatic analyzers under standard operating procedures approved for clinical use. The preoperative blood results (complete blood counts) of each patient were identified, and those closest to the time of surgery were recorded. Lymphopenia was indicated by a total lymphocyte count of <1,000/μl (19).

### Enumeration of Major Leukocyte Populations

Blood was collected in 4 ml EDTA-treated tubes (BD Biosciences, California, USA). Flow cytometry was performed on whole blood within 4 h after blood collection. Absolute numbers of T and B lymphocytes were quantified using TruCount tubes (BD Biosciences, California, USA). Absolute counts of CD45^+^ cells, CD3^+^, CD4^+^, CD8^+^ T lymphocytes and CD19^+^ B lymphocytes were analyzed on a 5-color BD TruCount flow cytometric assay, as described previously (20).

### Flow Cytometry

To measure apoptosis by flow cytometry, we stained the cells with Annexin V and propidium iodide (PI) (BD Biosciences) in order to estimate the rate of early-phase apoptosis, late-phase apoptosis or necrosis in each sample. PI indicates late apoptosis or necrotic cells. Cells that are positive for Annexin V, but not for PI are considered to be in early-phase apoptosis (21). To this end, cells were washed twice with cold PBS (Gibco) and resuspended at a concentration of 1 × 10^6^ cells/ml in Annexin V-binding buffer (BD Biosciences, San Jose, California, USA). They were then incubated for 15 min at room temperature with 5 μl Annexin V and 5 μl PI. 400 μl of Annexin V-binding buffer was then added and samples analyzed on a FACS Calibur cytometer (Cytek) using FlowJo v10 (Tree Star Corp) software. Every measurement includes 10^4^ cells.

To detect apoptosis, PBMCs were isolated and immediately resuspended at 1 × 10^6^ cells/ml. Multicolor cytofluorimetric analysis was then done using CD3-PE/cy7, CD4-percp-cy5.5, CD8-BV510, CD19-APC (all from BioLegend) antibodies. This analysis was done by automatic compensation for minimized fluorescence spillover and by using fluorescence minus one (FMO) control to establish positive/negative boundaries.

To detect pyroptosis, PBMCs were stained with CD3-PE/cy7, CD4-percp-cy5.5, CD8-BV510 and CD19-APC (all from BioLegend) antibodies. They were then incubated with fluorochrome-labeled Caspase-1 Inhibitors (Immunohistochemistry Technologies), which irreversibly bind to activated caspase-1.

### Statistical Analysis

Patient clinical characteristics and postoperative outcomes were presented as frequencies and percentages for categorical variables and compared using chi-square test or Fisher exact test. Normally distributed continuous variables were presented as mean and standard deviations (SD) and compared using Student *t* test while non-normally distributed variables were presented as medians and interquartile 25th and 75th percentiles (IQRs) and compared using the non-parametric Mann-Whitney U tests. Survival curves within time in ventilation, length in ICU stay and hospital stay were plotted by the Kaplan Meier (KM) method and compared by log-rank test. For the retrospective analysis, multivariable logistic regression was used to evaluate the independent predictive value of pre-absolute lymphocyte count (ALC) in primary study endpoints and to adjust for possible confounding factors. Variables with *p* < 0.10 from univariable analyses results were considered confounders in the multivariable logistic regression analysis. Results of the logistic regression model are given as odds ratio (OR) and 95% confidence interval (CI). For the prospective cohort, CD4^+^ T cells counts as a predictor for postoperative outcomes were estimated by receiver operating characteristic (ROC) curve analysis. Youden's index was defined for all the points along the ROC curve, and the maximum value of the index was used as a criterion for selecting the optimum cut-off point. The ability of the cut-off value for CD4^+^ T cells counts to predict postoperative outcomes were further evaluated by using multivariable logistic regression analysis. We hypothesized that the area under the curve of the MAE of CD4^+^ T cells counts would be 0.8. We calculated the required sample size for the ROC analysis. Considering the α error of 0.05, 90% power, and sample size ratio in the negative/positive group of 1, among MAEs and No MAEs, 34 patients were needed. Considering the attrition rate of 10%, at least a total of 38 AAD patients were included in the study. Actually, 40 AAD patients were included in our study. Linear regression was used to analyze the influence of apoptosis rate of lymphocyte (CD3^+^, CD4^+^ and CD8^+^ T cells) on their absolute counts. Data analysis was done using SPSS 23.0 software (SPSS, Chicago, IL, USA). All tests were two-sided and considered statistically significant at *p* < 0.05.

## Results

### Clinical Characteristics of the Patients

Clinical baseline characteristics in AAD are shown in Table 1. Demographic characteristics of lymphopenia and non-lymphopenia populations were similar in terms of pre-operative ejection fraction (EF) value, serum creatinine levels, hypothermic circulatory arrest (HCA) time, HCA temperature, aortic cross clamp time (ACCT), CPB time and comorbidities. Male gender and smoking status were more frequent among the group with non-lymphopenia (*p* < 0.05). Relative to non-lymphopenia patients, lymphopenia correlated with more advanced age, longer time of operation (*p* < 0.05) and more percentage of coronary heart disease (13 vs. 6%; *p* = 0.028). The percentage of lymphopenia patients undergoing coronary artery bypass grafting (CABG) surgery (13 vs. 4%; *p* = 0.011) and total arch replacement (98 vs. 91%; *p* = 0.014) was higher than non-lymphopenia patients. Other significant differences included shorter time of symptom onset to hospital presentation (12 vs. 21 h; *p* < 0.001) and lower pre-operative monocyte count (0.6 vs. 0.8 × 10^9^/L; *p* < 0.001).

**Table 1:** Demographic and clinical characteristics of patients with lymphopenia and non-lymphopenia.

	**Lymphopenia (*n* = 167)**	**Non-lymphopenia (*n* = 128)**	***p- value***
**Demographics**			
Age	52 ± 9.0	48 ± 10	0.004
Male	125 (75)	111 (87)	0.012
**Debakey classification**			0.015
I	167 (100)	123 (96)	
II	0 (0)	5 (4)	
**Medical history**			
Smoking	66 (40)	67 (52)	0.028
Hypertension	123 (74)	97 (76)	0.677
Diabetes mellitus	6 (4)	3 (2)	0.783
Marfan syndrome	3 (3)	2 (2)	1.000
Coronary heart disease	22 (13)	7 (6)	0.028
Cardiovascular surgery	4 (2)	3 (2)	1.000
Cerebral vascular disease	10 (6)	4 (3)	0.384
Organ malperfusion	73 (44)	53 (41)	0.691
**Laboratory results**			
WBC, 10^9^/L	12.4 ± 3.5	13.2 ± 4.0	0.064
Neutrophil, 10^9^/L	10.6 (8.6, 12.6)	10.5 (8.1, 13.1)	0.578
Monocyte, 10^9^/L	0.6 (0.5, 0.8)	0.8 (0.7, 1.1)	<0.001
Lymphocyte, 10^9^/L	0.8 (0.6, 0.9)	1.3 (1.1, 1.5)	<0.001
Creatinine, mg/dL	1.0 (0.8, 1.3)	0.9 (0.8, 1.4)	0.964
LVEF, %	66 (62, 70)	65 (61, 69)	0.833
Symptom onset to hospital presentation, h	12 (8, 24)	21 (12, 39)	<0.001
Presentation to surgery, h	22 (13, 35)	21 (14, 35)	0.580
**Procedure type**			
Total arch replacement	164 (98)	117 (91)	0.014
Hemiarch replacement	2 (1)	6 (5)	0.142
Bentall procedure	7 (4)	5 (4)	1.000
David procedure	7 (4)	5 (4)	1.000
CABG	22 (13)	5 (4)	0.011
**Duration of procedure**			
CPB, h	3.8 (2.8, 4.7)	3.4 (2.3, 4.6)	0.052
ACCT, h	1.7 (1.0, 2.5)	1.4 (0.9, 2.3)	0.098
HCA ≥ 30 min	47 (28)	29 (23)	0.285
HCA temperature, °C	25.3 (24, 28.4)	27 (24.8, 29)	0.070
Duration of surgery, h	8.5 ± 2.4	7.8 ± 2.5	0.032

*Data are presented as number (%), mean (SD) or median (IQR)*.

*Depending on the types of data, the Student t test or Mann-Whitney test or Fisher exact test was applied, and p < 0.05 was considered to indicate statistical significance*.

*ACCT, aortic cross clamp time; CABG, coronary artery bypass grafting; CPB, cardiopulmonary bypass time; HCA, hypothermic circulatory arrest; LVEF, left ventricular ejection fraction; IQR, interquartile range; WBC, white blood cell*.

### Lymphopenia Predicts Post-operative Complications

Analysis of correlation between lymphopenia and postoperative MAEs revealed significantly higher rates of AKI, infection, arrhythmia, myocardial infarction, mortality, and overall MAEs in lymphopenia patients relative to non-lymphopenia group (*p* < 0.05, Table 2). 37% of the lymphopenia patients developed AKI after surgery, compared to17% in controls (*p* < 0.001). Mortality rate was higher in the lymphopenia relative to non-lymphopenia group (10 vs. 5%; *p* = 0.042). No difference was observed in lymphopenia patients relative to non-lymphopenia group in terms of cerebrovascular accident, spinal cord injury, re-intubation, and re-operation (Table 2).

**Table 2:** Postoperative events of patients with lymphopenia and non-lymphopenia.

	**Lymphopenia (*n* = 167)**	**Non-lymphopenia (*n* = 128)**	***p- value***
AKI	61 (37)	22 (17)	<0.001
Infection	64 (38)	34 (27)	0.034
Arrhythmia	55 (33)	19 (15)	<0.001
Re-operation	11 (7)	4 (3)	0.283
Re-intubation	19 (11)	8 (6)	0.120
Spinal cord injury	20 (12)	10 (8)	0.241
Cerebrovascular accident	39 (23)	28 (22)	0.764
Postoperative myocardial infarction	14 (8)	2 (2)	0.021
Mortality	17 (10)	5 (5)	0.042
MAE	128 (77)	60 (47)	<0.001

*Data are presented as number (%)*.

*χ^2^ test or Fisher exact test was applied to examine postoperative events of patients with lymphopenia and non-lymphopenia. The p < 0.05 was considered to indicate statistical significance*.

*AKI, acute kidney injury; MAE, major adverse events*.

Multivariable analysis showed that pre-operative lymphopenia independently correlated with increased risk of MAE after surgery (OR, 4.152; 95% CI 2.434–7.081; *p* < 0.001) (Tables 3, 4). Other predictors include organ malperfusion (OR, 2.481; 95% CI 1.432–4.298; *p* = 0.001), and CPB time (OR, 1.285; 95% CI, 1.063–1.552; *p* = 0.010). As shown in Supplementary Figure 3, KM curves indicated patients with lymphopenia had longer time in ventilation during a 72-h followed-up periods (*p* = 0.001), more length of ICU stay (*p* < 0.001) and hospitalizations (*p* = 0.005).

**Table 3:** Baseline characteristics of patients with MAE and no MAE in retrospective cohort.

	**MAE (*n* = 188)**	**No MAE (*n* = 107)**	***p-value***
**Demographics**			
Age	51 (46, 56)	49 (42, 55)	0.009
Male	150 (80)	86 (80)	0.904
**Debakey classification**			0.357
I	186 (99)	104 (97)	
II	2 (1)	3 (3)	
**Medical history**			
Smoking	88 (47)	45 (42)	0.430
Diabetes	6 (3)	3 (3)	1.000
Marfan syndrome	3 (2)	2 (2)	1.000
Hypertension	145 (77)	75 (70)	0.182
Coronary heart disease	24 (13)	5 (5)	0.025
Cardiovascular surgery	4 (2)	3 (3)	0.707
Cerebral vascular disease	11 (6)	3 (3)	0.369
Organ malperfusion	93 (50)	33 (31)	0.002
**Laboratory results**			
WBC, 10^9^ /L	12.4 (10, 15.3)	9.8 (12.4, 14.1)	0.413
Neutrophil, 10^9^ /L	10.6 (8.5, 13.2)	10.4 (8.1, 11.9)	0.085
Monocyte, 10^9^ /L	0.7 (0.5, 0.9)	0.8 (0.6, 1.0)	0.179
Lymphocyte, 10^9^ /L	0.9 (0.7, 1.1)	1.1 (0.9, 1.4)	<0.001
Creatinine, mg /dL	1.0 (0.8, 1.5)	0.9(0.7, 1.2)	0.006
LVEF, %	66 (61, 70)	65 (61, 69)	0.849
Symptom onset to hospital presentation, h	13 (6, 21)	18 (7, 30)	0.075
Presentation to surgery, h	21 (11, 32)	25 (14, 36)	0.012
**Involvement of vessel branches**			
Coronary artery	63 (33)	30 (28)	0.331
Innominate artery	119 (63)	52 (49)	0.014
LSA	102 (54)	50 (47)	0.214
Left common carotid artery	96 (51)	49 (46)	0.384
Celiac trunk	71 (38)	34 (32)	0.302
Superior mesenteric artery	48 (26)	22 (21)	0.335
Right renal artery	56 (30)	31 (29)	0.883
Left renal artery	76 (40)	38 (36)	0.405
**Procedure type**			
Total arch replacement	179 (95)	102 (95)	0.965
Hemiarch replacement	7 (4)	1 (1)	0.296
Bentall procedure	9 (5)	3 (3)	0.601
David procedure	8 (4)	4 (4)	1.000
CABG	23 (12)	4 (4)	0.026
**Duration of procedure**			
CPB, h	3.7 (2.8, 4.9)	3.5 (2.1, 4.3)	0.001
ACCT, h	1.8 (1.0, 2.6)	1.3 (0.7, 2.2)	0.001
HCA ≥ 30 min	54 (29)	22 (21)	0.123
HCA temperature, °C	25.2 (24.3, 29)	27.6 (25, 29)	0.040
Time of surgery, h	8.7 (6.8, 10.1)	7.7 (5.5, 8.8)	<0.001

*Values are expressed as number (%), mean (SD) or median (IQR)*.

*Depending on the types of data, the Student t test or Mann-Whitney test or Fisher exact test was applied, and p < 0.05 was considered to indicate statistical significance*.

*ACCT, aortic cross clamp time; CABG, coronary artery bypass grafting; CPB, cardiopulmonary bypass time; HCA, hypothermic circulatory arrest; LSA, left subclavian artery; LVEF, left ventricular ejection fraction; MAE, Major adverse events; IQR, interquartile range; WBC, white blood cell*.

**Table 4:** Univariable and multivariable logistic regression analysis of possible predictors of MAE.

**Predictors**	**Univariable**		**Multivariable**	
	**OR (95% CI)**	***p-value***	**OR (95% CI)**	***p-value***
Age, years	1.028 (1.003–1.054)	0.027		
Coronary heart disease	2.985 (1.204–8.072)	0.031		
Innominate artery injury	1.824 (1.127–2.952)	0.014		
Organ malperfusion	2.195 (1.331–3.620)	0.002	2.481 (1.432–4.298)	0.001
CABG surgery	3.589 (1.207–10.675)	0.022		
Symptom onset to hospital presentation, hour	0.993 (0.986–1.000)	0.047		
Creatinine, mg/dL	1.581 (0.989–2.529)	0.056		
Neutrophil count, 10^9^ /L	1.071 (0.998–1.149)	0.057		
Lymphopenia	3.970 (2.404–6.556)	<0.001	4.152 (2.434–7.081)	<0.001
CPB, h	1.384 (1.155–1.659)	<0.001	1.285 (1.063–1.552)	0.010
ACCT, h	1.579 (1.199–2.080)	0.001		
HCA temperature, °C	0.930 (0.859–1.007)	0.074		

*Binary logistic regression analysis was applied to examine factors associated with the MAE (dependent variable) in the retrospective cohort (n = 295). The p < 0.05 was considered to indicate statistical significance*.

*ACCT, aortic cross clamp time; CABG, coronary artery bypass grafting; CPB, cardiopulmonary bypass time; CI, confidence interval; HCA, hypothermic circulatory arrest; MAE, major adverse events; OR, odds ratio*.

### CD4^+^ T Lymphopenia Correlates With MAEs After AAD Surgery

**Figure 1:**
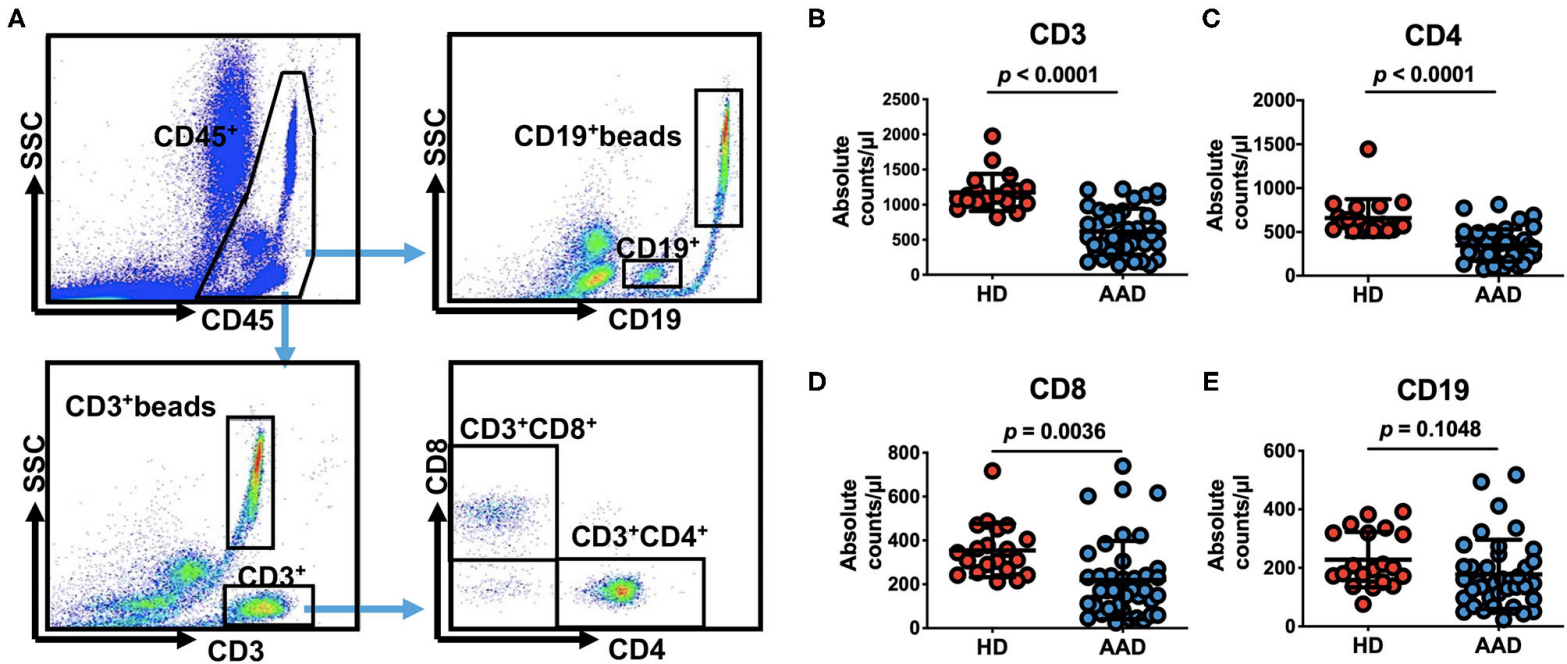
Pre-operative peripheral blood lymphocyte subsets count in 40 patients undergoing surgical repair of type A aortic dissection and 20 healthy donors. **(A)** Quantitation of numbers in a lysed whole-blood, that is, all lymphocytes presented in the lymphocyte gate identified by CD45 fluorescence and side scatter on flow cytometry and consisting mainly of CD3^+^, CD4^+^, CD8^+^, CD19^+^ cells and their corresponding beads are examined. **(B–E)** Comparison of pre-operative peripheral blood lymphocyte subsets counts between AAD patients and HDs (data shown as means ± SD). CD3^+^ beads, the beads corresponding CD3^+^ cells, CD19^+^ beads, the beads corresponding CD19^+^ cells.

Next, we sought to identify the lymphocyte subgroups that correlate with clinically significant outcomes. We found that lymphopenia in AAD patients is primarily due to the reduction of T cells (Figure 1). CD3^+^ T cells counts, but not B cells, were significantly reduced in the AAD group compared to healthy donors (HDs) (CD3^+^ T cells: 615.6 ± 327.3 vs. 1,175.0 ± 264.3 cells/μl, *p* < 0.0001; CD19^+^ B cells: 177.8 ± 118.3 vs. 228.3 ± 94.5 cells/μl, *p* = 0.1048). Among CD3^+^ T cells, reduced CD4^+^ and CD8^+^ T cells were observed in the AAD group as compared with HD group (CD4: 346.7 ± 183.6 vs. 659.0 ± 214.6 cells/μl, *p* < 0.0001; CD8: 219.5 ± 178.4 vs. 354.4 ± 121.8 cells/μl, *p* = 0.0036) (Figure 1).

**Figure 2:**
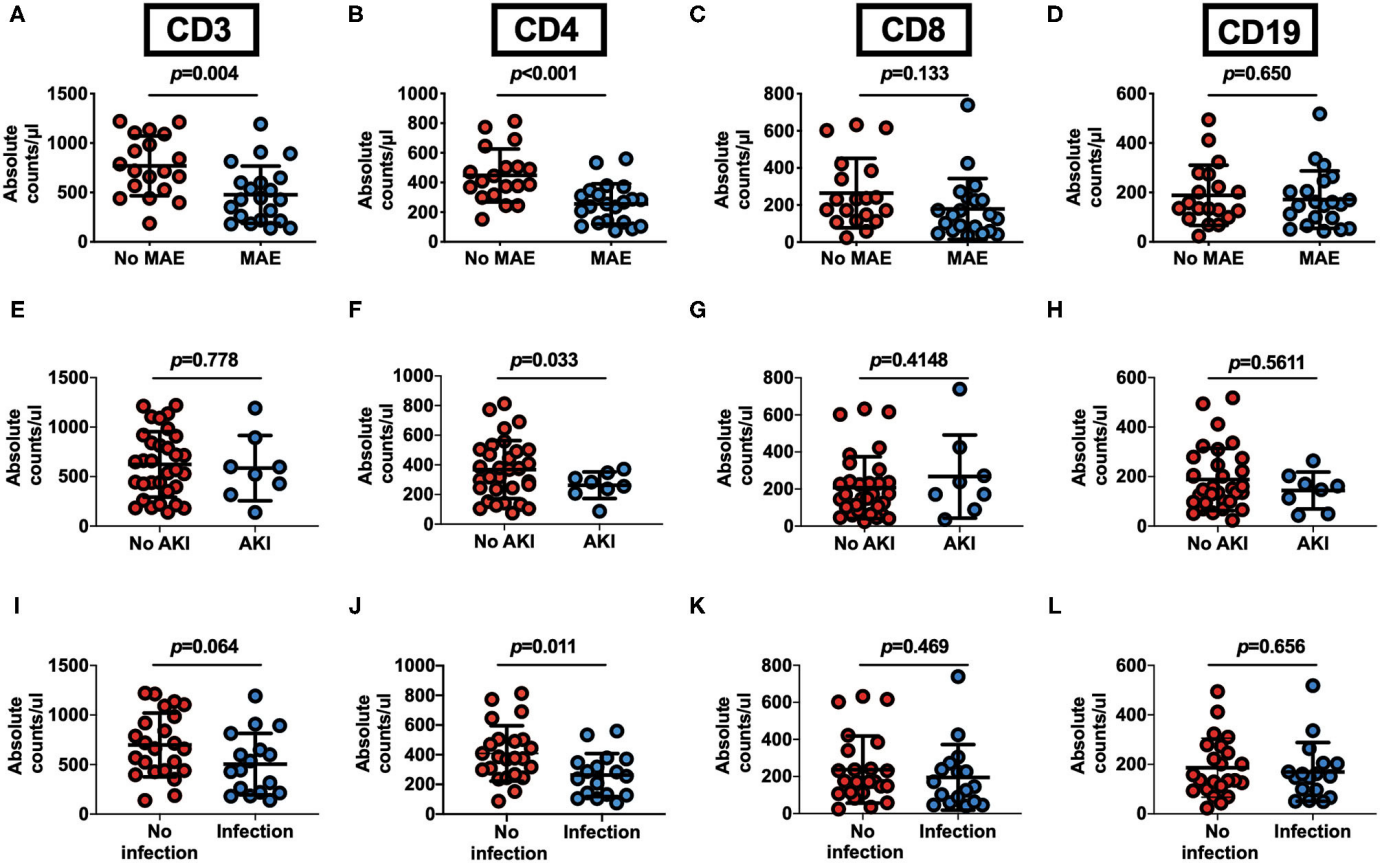
Pre-operative peripheral blood CD4^+^ T lymphocyte count as an outcome predictor following surgery for acute Type A aortic dissection. **(A–D)** Pre-operative peripheral blood lymphocyte subsets counts in 40 patients undergoing surgical repair of type A aortic dissection with respect to postoperative occurrence of MAEs. AAD patients with MAE (*n* = 21) or no MAE (*n* = 19). **(E–H)** Preoperative peripheral blood lymphocyte subsets counts in AAD patients with or without postoperative AKI. Patients with AKI (*n* = 8) and No AKI (*n* = 32). **(I–L)** Pre-operative peripheral blood cells lymphocyte subsets counts in AAD patients with or without postoperative infection. Patients with infection (*n* = 17) and No infection (*n* = 23). Plots indicate means ± SD.

When stratified according to MAEs incidence, lymphocyte subset analysis showed that patients who developed complications had lower preoperative CD3^+^ and CD4^+^ T cells levels relative to those with an uneventful recovery (CD3^+^ T cells: 768.9 ± 302.4 vs. 476.9 ± 289.9 cells/μl, *p* = 0.0035; CD4^+^ T cells: 447.6 ± 179.0 vs. 255.4 ± 135.8 cells/μl, *p* = 0.004, No MAE group vs. MAE group) (Figures 2A,B). In contrast, CD8^+^ T cells and B cells did not differ between no MAE group and MAE group (CD8^+^ T cells: 264.3 ± 187.3 vs. 178.9 ± 163.9 cells/μl, *p* = 0.1326; CD19^+^ B cells: 188.3 ± 122.1 vs. 171 ± 116.2 cells/μl, *p* = 0.6505) (Figures 2C,D). Similar observations show CD4^+^ T cells levels relative to those with AKI and infection (Figures 2E–L). In addition, we found that the pre-operative CD4^+^ T cells counts at a cut-off value of 357.96 cells/μl is an effective and reliable predictor of MAEs (area under ROC curve 0.817; 95% CI, 0.684-0.950; *p* < 0.005). This cut-off achieves a 74% sensitivity and 81% specificity, supporting the hypothesis that CD4^+^ T lymphopenia may predispose to poor AAD surgical outcomes (Supplementary Figure 4). After adjustment for age, organ malperfusion, symptom onset to surgery, multivariate analysis revealed CD4^+^ T cells count as being independently correlated with elevated MAEs (HR, 9.384; 95% CI 1.85–47.59; *p* = *0.009)* (Table 5; Supplementary Table 2).

**Table 5:** Univariable and multivariable logistic regression analysis of possible predictors of MAE in prospective cohort.

**Predictors**	**Univariable**		**Multivariable**	
	**OR (95% CI)**	***p-value***	**OR (95% CI)**	***p-value***
Age, years	1.028 (0.969–1.096)	0.337		
CD4^+^ T cells lymphopenia	11.90 (2.674–52.96)	0.001	9.384 (1.850– 47.59)	0.009
Organ malperfusion	16.36 (1.835–150.0)	0.012	11.90 (1.123–126.2)	0.040
Symptom onset to surgery, h	0.993 (0.986–1.001)	0.091		

*Binary logistic regression analysis was applied to examine factors associated with the MAE (dependent variable) in the prospective cohort (n = 40). The p < 0.05 was considered to indicate statistical significance*.

*CI, confidence interval; MAE, major adverse events; OR, odds ratio*.

### Apoptosis, but Not Pyroptosis Contributes to CD4^+^ T Lymphopenia in AAD

**Figure 3:**
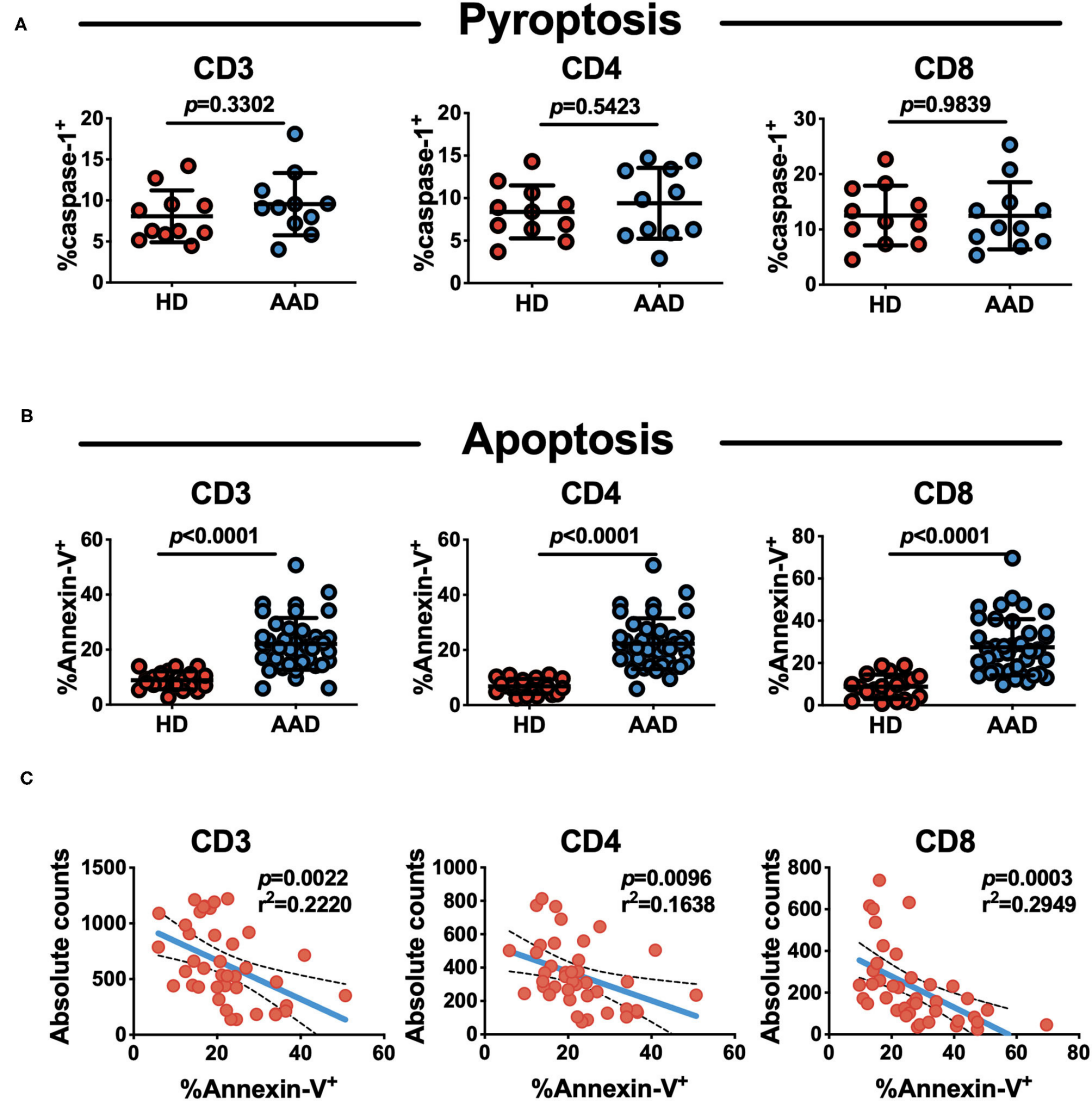
Increased spontaneous apoptosis, not pyroptosis of T lymphocytes in AAD patients. **(A)** Analysis of pyroptosis status of lymphocyte subsets with flow cytometry (*n* = 11). **(B)** Analysis of apoptosis status of lymphocyte subsets with flow cytometry. Mean ± SD comparing their rates on AAD patients (*n* = 40) and HDs (*n* = 20). **(C)** Correlation of absolute lymphocyte (CD3^+^, CD4^+^ and CD8^+^) counts and apoptosis rate in AAD patients (*n* = 40).

We assessed the spontaneous cell death in AAD patients. Pyroptosis and apoptosis are two major types of active cell death. This analysis did not reveal differences between AAD patients and healthy subjects in terms of lymphocyte pyroptosis (Figure 3A). Of note, lymphocytes undergoing apoptosis were markedly elevated in AAD patients relative to those in healthy subjects (Figure 3B). The apoptosis rate of AAD T lymphocytes was significantly higher relative to that of healthy subjects (8.100 ± 3.958 vs. 22.12 ± 9.512%, *p* < 0.0001). Separate analysis of changes in T cells subsets, revealed that AAD patients exhibited significantly higher CD4^+^ (6.243 ± 3.168 vs. 20.010 ± 9.054%, *p* < 0.0001) and CD8^+^ T cells (8.003 ± 5.963 vs. 23.720 ± 12.920%, *p* < 0.0001) apoptosis relative to healthy subjects. Correlation analysis showed that CD4^+^ and CD8^+^ T lymphocytes apoptotic rates were inversely correlated to their absolute counts (Figure 3C).

## Discussion

There is a high incidence of postoperative complications in AAD patients undergoing surgery (22). In the present study, we have shown that pre-operative lymphopenia is predictive of poor outcome after AAD surgery. Lymphopenia is primarily driven by loss of CD4^+^ and CD8^+^ T cells subsets, which result from spontaneous apoptosis but not pyroptosis. Furthermore, we have identified an association between CD4^+^ T cells population and development of MAEs, with the AUC of ROC analyses for distinguishing between MAE and no MAE subjects to be 0.8. This provides what we believe to be the first evidence of a role for these cells in AAD prognosis after surgery.

Inflammatory response has been known as key mechanism underlying aortic dissection. The upregulation of cytokines, endothelial adhesion molecules or chemokines are found to play a role in multiple organ injury, including myocardial ischemic injury, acute kidney injury and gastrointestinal disorders (23–28). These data led us to speculate that immune cells are important to the postoperative organ injury in AAD patients. However, current risk assessment indices for AAD outcomes do not incorporate leukocyte subsets despite their close correlation with infection and heart failure (29, 30). The present study is the first, to our knowledge, to directly assess the prognostic significance of lymphocyte counts alone in AAD patients after surgery. The overall prevalence of lymphopenia observed in our study was markedly high (56.6%). Our data implicate that decreased lymphocytes, but not altered monocytes or neutrophils, are closely associated with postoperative MAEs, including acute renal injury, infection, myocardial infarction, arrhythmia, and death during hospitalization.

Our data also show that lymphocyte loss is primarily due to the reduced CD4^+^ and CD8^+^ T cells, but not CD19^+^ B cells. The decrease of CD4^+^ T cells in AAD patients is consistent with Porto's report (23). But a reduction of CD8^+^ T cells and no significant change of B cells were found in the present study, in contrast to Porto' report delineating an increase in B cells and unchanged CD8^+^ T cells in AAD patients. This discrepancy may be due to the different detection methods. Porto' study used the percentage of CD8^+^ T cells or B cells against total lymphocytes, unlike absolute numbers of T and B lymphocytes quantified using TruCount tubes in the present study. Given the dramatic reduction of total lymphocytes counts in most of AAD patients, the fact that unchanged B cells absolute number divided by decreased total lymphocytes counts makes the percentage of B cells higher than healthy controls, so did CD8^+^ T cells. Together with Porto's study, our observations suggest a more profound reduction of CD4^+^ T cells than that of CD8^+^ T cells.

Lymphocytes pyroptosis and apoptosis are widely reported in various diseases (31–33). However, we observed that lymphocyte pyroptosis in AAD patients was comparable to that of healthy subjects, whereas lymphocyte apoptosis was dramatically increased in AAD patients. These findings indicate that apoptosis, but not pyroptosis, may at least in part, account for lymphocyte loss. Indeed, the apoptotic rate of CD4^+^ T cells was inversely related with absolute lymphocyte counts. Apoptosis may be triggered during early AAD stages, when intense inflammation causes severe tear of the aortic intima or when ischemia reperfusion of multi-organs stimulates release of pro-apoptotic substances, such as TNF-α and nitric oxide. Supporting this assumption, our recent study showed the serum derived from AAD patients activated gene expression of the pro-inflammatory cytokines in the cultured peripheral blood mononuclear cells from HDs (34).

Importantly, CD4^+^ T lymphopenia is correlated with postoperative MAEs in the present study. It is interesting to note the dramatic exhaustion of lymphocytes caused by apoptosis. The apoptosis of T lymphocytes, in turn, may contribute to immunosuppression through the effects of apoptotic cells. In fact, except for lymphocyte apoptosis, decreased HLA-DR expression was also reported in AAD patients after surgery (35). These findings suggest that intense and constant tear of aortic wall in AAD may cause an immunosuppressive state like sepsis, which substantially contributes to morbidity and mortality. Indeed, sepsis induced immunosuppression is well-known characterized by lymphocyte exhaustion and the reprogramming of antigen-presenting cells (36). In addition, acute loss of lymphocytes from circulating blood also occurs following ischemia-reperfusion in STEMI patients receiving PPCI, and the lymphopenia after PPCI predicts long-term mortality in STEMI patients (37). CD4^+^ T cells depletion may promote poor outcomes via multiple mechanisms, including weakened immunity, disruption of the balance between anti-inflammatory and pro-inflammatory mediators, and exacerbation of myocardial damage. Despite the unclear mechanism, it is possible that T cell lymphopenia disrupts helper T cell (Th) 1/Th2 imbalance and reduces the population of regulatory T cells, which may accelerate recovery (38, 39).

## Limitations

A limitation of this study is difficulty in establishing causality in human subjects. Causality may be addressed by blocking and reconstituting effects in animal models, or through restoring the lymphocyte counts in randomized trials. Thus, our study only provides informative evidence and advances our understanding of the human immune system in clinical AAD surgery settings. Further investigations are needed to establish how specific CD4^+^ T cells subsets contribute to MAEs in AAD.

## Conclusion

In summary, our results highlight the prognostic value of preoperative lymphocyte counts in AAD patients undergoing surgery. In particular, the loss of CD4^+^ T cells via apoptosis may influence development of postoperative MAEs in AAD patients.

## Data Availability Statement

The raw data supporting the conclusions of this article will be made available by the authors, without undue reservation.

## Ethics Statement

The studies involving human participants were reviewed and approved by the Institutional Medical Ethics Review Board of the Second Xiangya Hospital, Changsha, China. The patients/participants provided their written informed consent to participate in this study.

## Author Contributions

HL and R-PD: concept, design, and critical revision of the manuscript for important intellectual content. DF, W-JL, and H-YZ: acquisition and analysis of data. J-JS, WL, and HL: conducting all the experiments, and drafting of the manuscript. J-JS, Z-LH, and HL: statistical analysis. HL, J-MX, and R-PD: administrative, technical, or material support. HL, R-PD, J-MX, and HT: supervision. All authors contributed to the article and approved the submitted version.

## Funding

This research was supported by the National Natural Science Foundation of China (NSFC 81873770 to HL, 82071347 and 81771354 to R-PD, and 81901231 to Z-LH) and the Natural Science Foundation of Hunan Province (2019JJ40445 to H-YZ).

## Conflict of Interest

The authors declare that the research was conducted in the absence of any commercial or financial relationships that could be construed as a potential conflict of interest.

## Publisher's Note

All claims expressed in this article are solely those of the authors and do not necessarily represent those of their affiliated organizations, or those of the publisher, the editors and the reviewers. Any product that may be evaluated in this article, or claim that may be made by its manufacturer, is not guaranteed or endorsed by the publisher.

## Supplementary Material

Supplementary Figure 1 Flow chart for retrospective cohort.

Supplementary Figure 2 Flow chart for prospective cohort.

Supplementary Figure 3 Kaplan–Meier analysis of the difference in the postoperative outcomes between lymphopenia and non-lymphopenia. Patients with lymphopenia had longer time in ventilation and more length of ICU stay and hospitalizations. We excluded the patients who died during the intensive care unit or hospitalization. **(A)** Mechanical ventilation for ≥ 72 h; **(B)** length of ICU stay for ≥14 days; **(C)** hospital stay ≥ 30 days. *P* values were generated by log-rank test.

Supplementary Figure 4 Receiver-operating characteristic curves displaying the ability of the lymphocyte count and CD4^+^ T cells count to predict postoperative outcomes in AAD patients in the prospective cohorts. AUC, the area under the curve; CI, confidence interval.

Supplementary Table 1 Baseline data of the enrolled AAD patients and control donors.

Supplementary Table 2 Baseline characteristics of patients with MAE and No MAE in prospective cohort.
