# Supplementary figures and images for: Association of Apoptosis-Mediated CD4+ T Lymphopenia With Poor Outcome After Type A Aortic Dissection Surgery
Source: Front Cardiovasc Med. 2021 Nov 12;8:747467. doi: 10.3389/fcvm.2021.747467 (PMC8632808; doi:10.3389/fcvm.2021.747467)

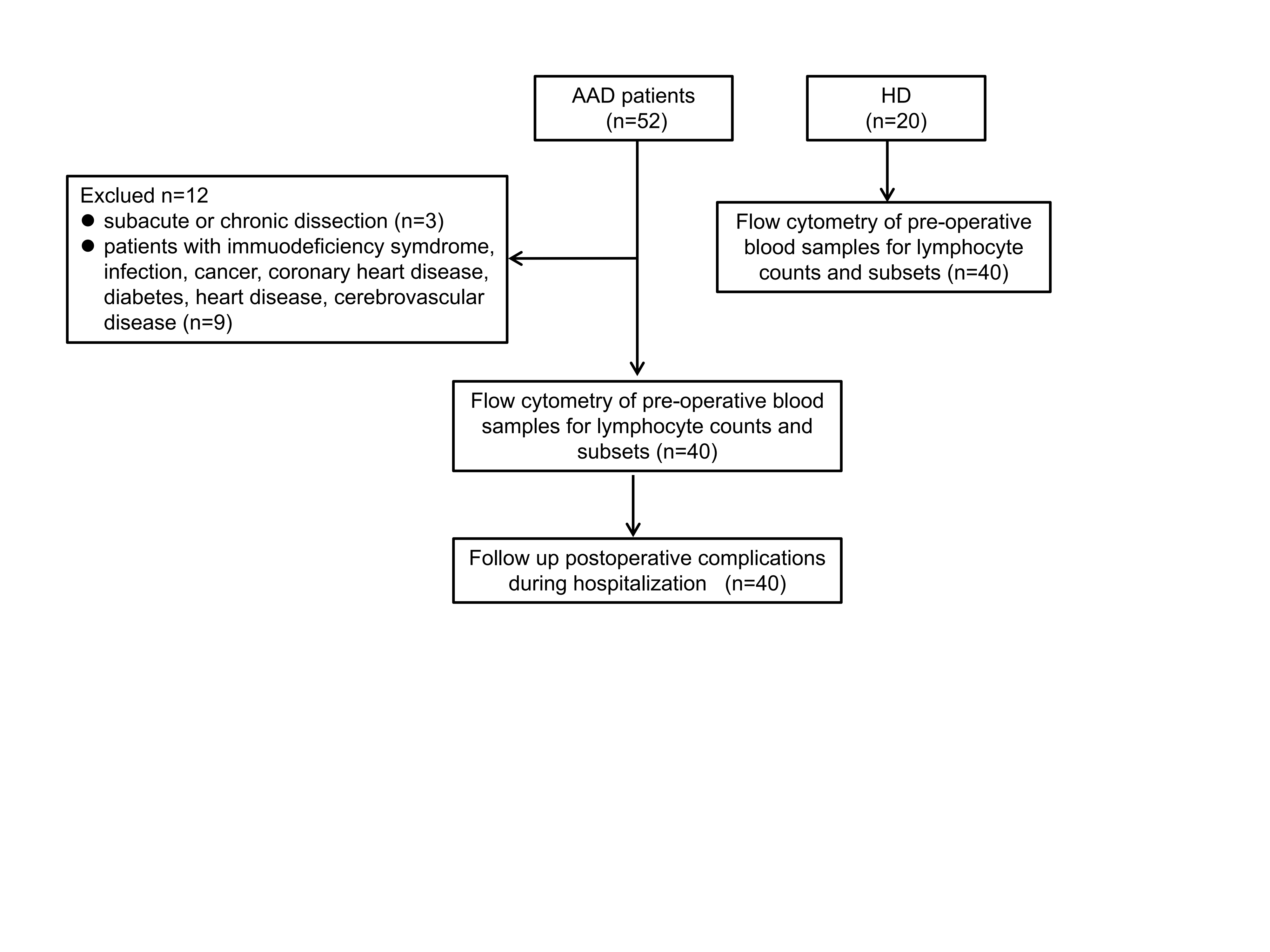

Supplement: Supplementary Figure 1 — Flow chart for retrospective cohort. [file Data_Sheet_1.zip › Figures/Supplemental Figure 2.tif]

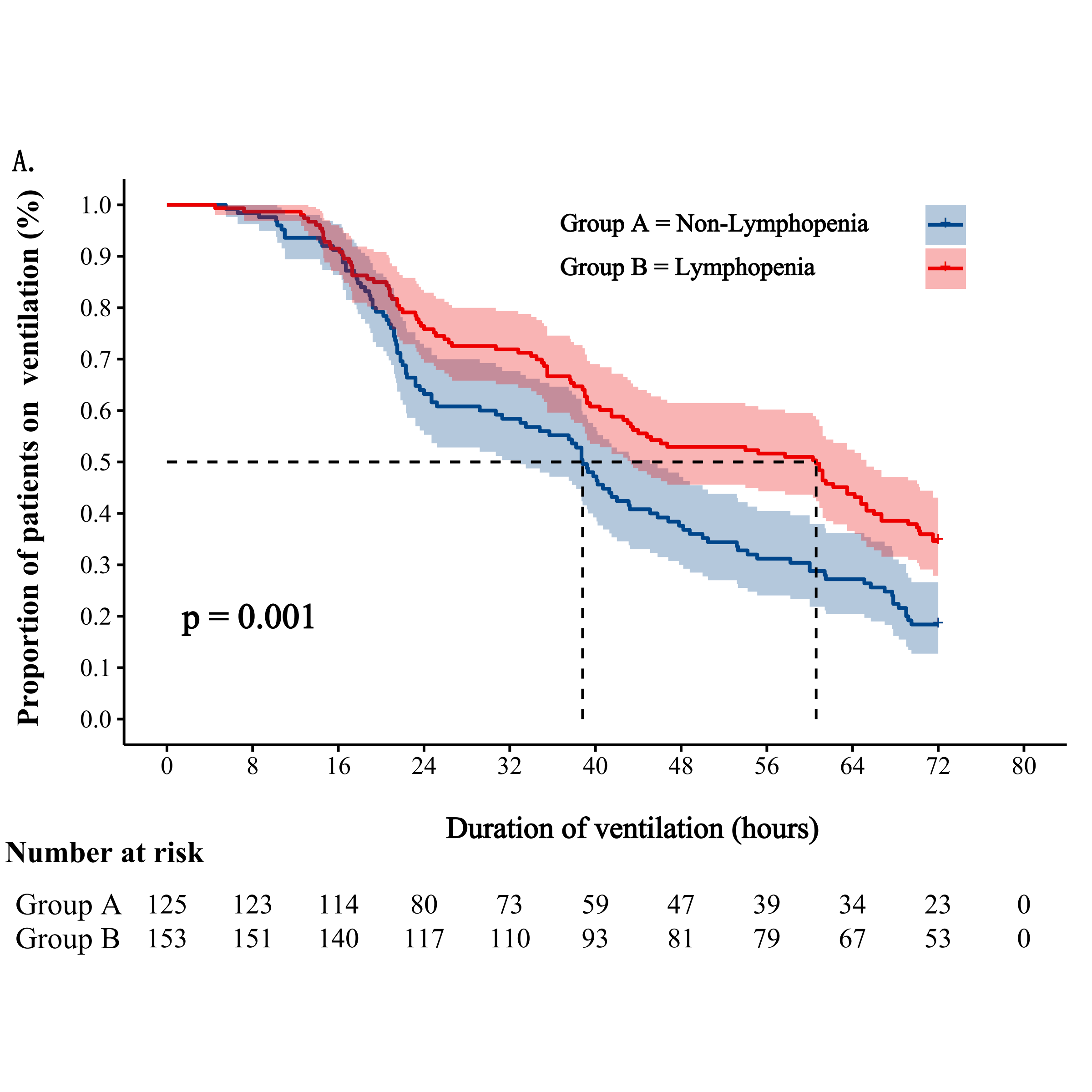

Supplement: Supplementary Figure 1 — Flow chart for retrospective cohort. [file Data_Sheet_1.zip › Figures/Supplemental Figure 3A.tif]

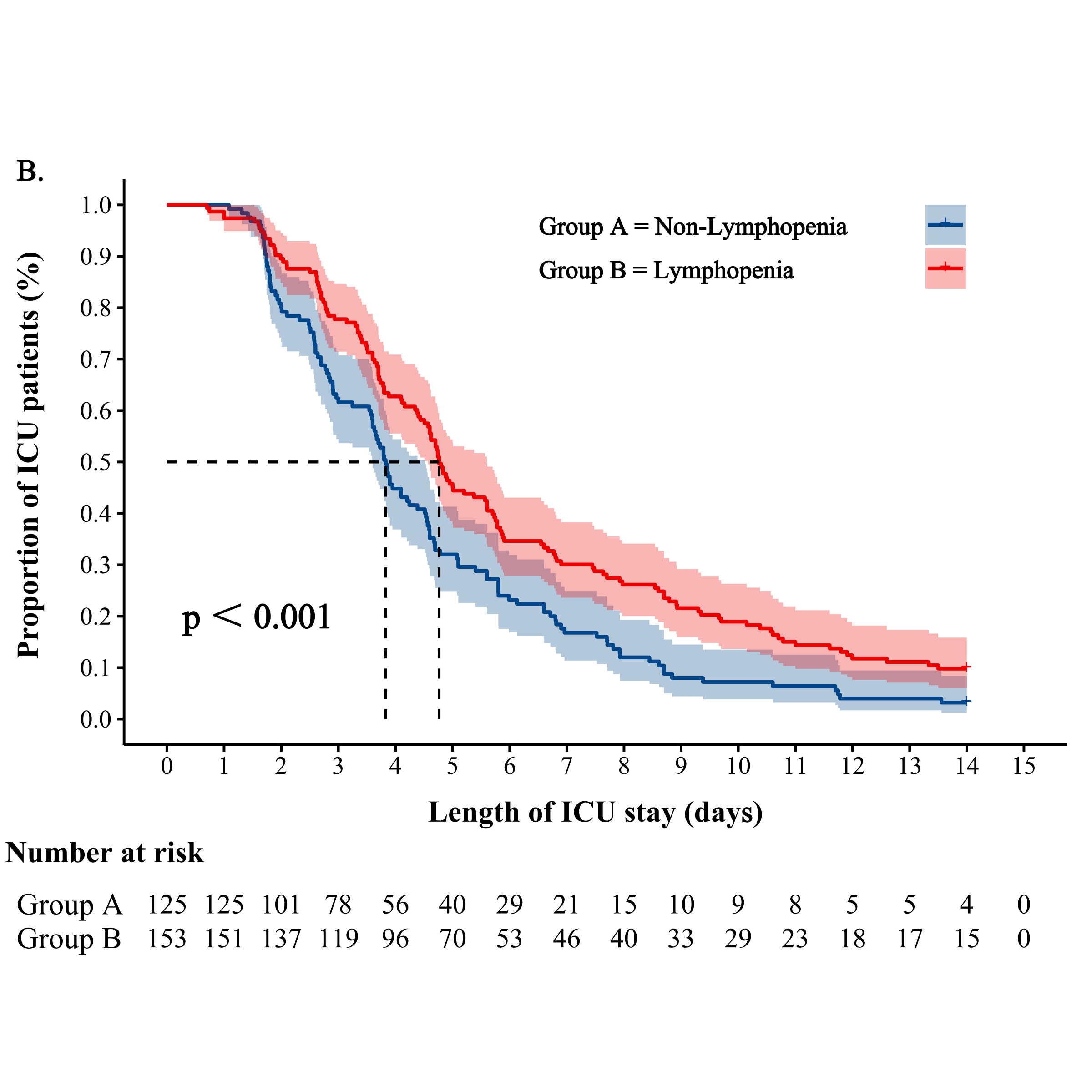

Supplement: Supplementary Figure 1 — Flow chart for retrospective cohort. [file Data_Sheet_1.zip › Figures/Supplemental Figure 3B.tif]

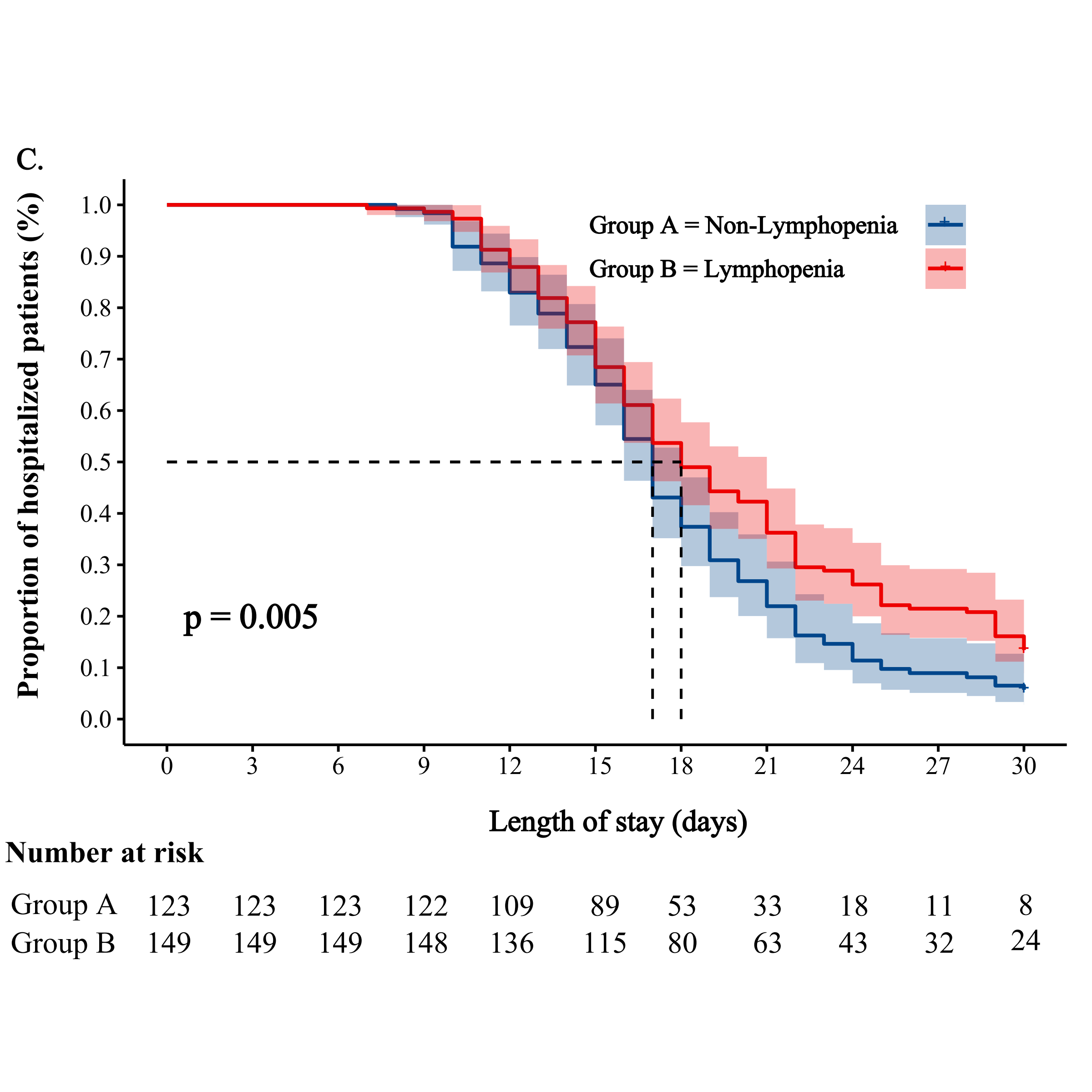

Supplement: Supplementary Figure 1 — Flow chart for retrospective cohort. [file Data_Sheet_1.zip › Figures/Supplemental Figure 3C.tif]

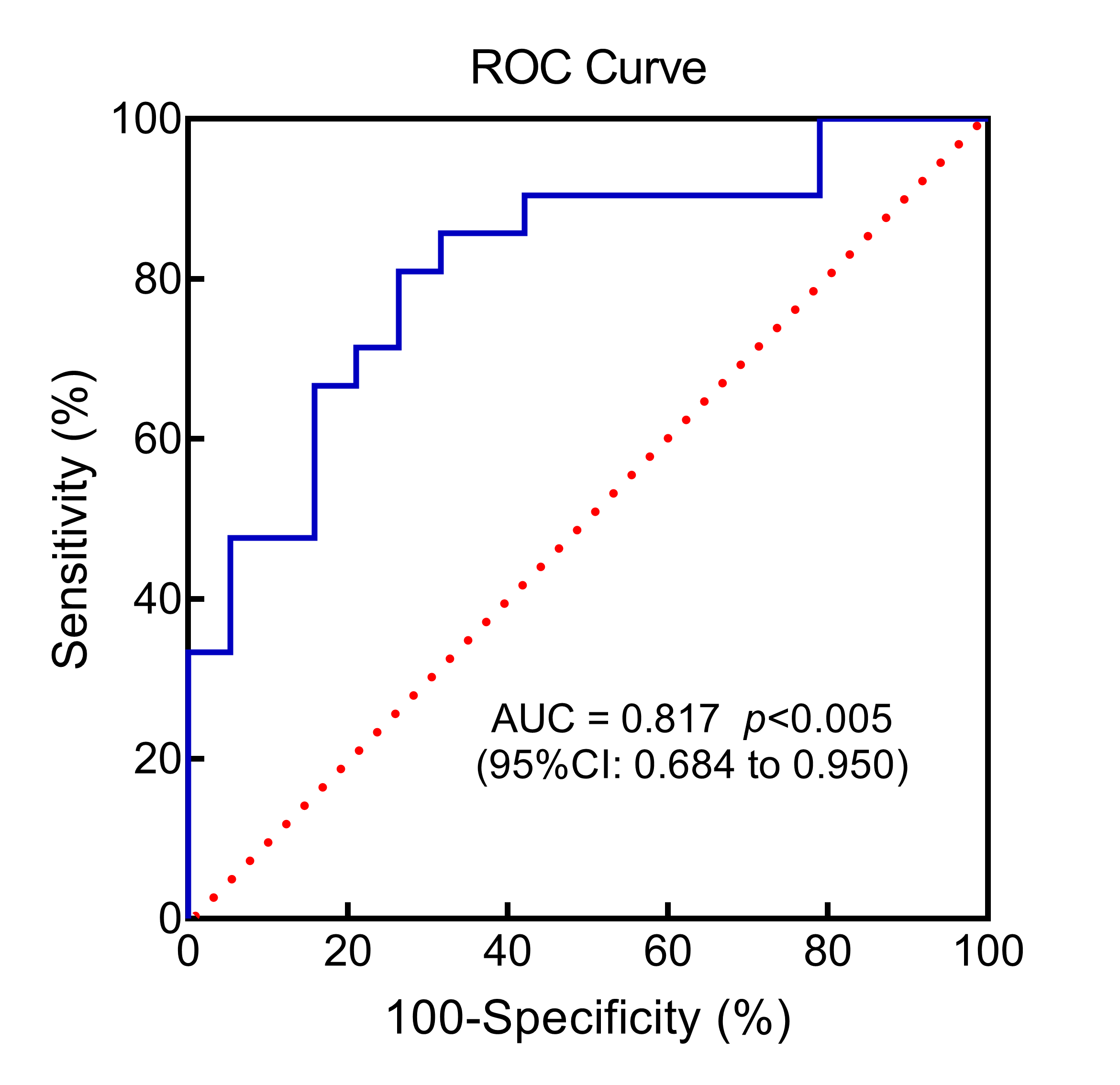

Supplement: Supplementary Figure 1 — Flow chart for retrospective cohort. [file Data_Sheet_1.zip › Figures/Supplemental Figure 4.tif]
