# Supplementary material for: Association of Apoptosis-Mediated CD4+ T Lymphopenia With Poor Outcome After Type A Aortic Dissection Surgery
Source: Front Cardiovasc Med. 2021 Nov 12;8:747467. doi: 10.3389/fcvm.2021.747467 (PMC8632808; doi:10.3389/fcvm.2021.747467)
Supplement: Supplementary Table 1 — Baseline data of the enrolled AAD patients and control donors. [file Table_1.docx]

**Supplemental Table1. Baseline data of the enrolled AAD patients and healthy donors**

| **Variables** | **HD** | **AAD** |
| --- | --- | --- |
| Number, n | 20 | 40 |
| Age | 54 ± 3 | 50 ± 2 |
| Male | 15 (75) | 32 (80) |
| Smoking | 0 (0) | 14 (35) |
| Hypertension | 0 (0) | 32 (80) |

Date are presented as n(%) or mean (SD)

AAD, acute aortic dissection; HD, healthy donors; SD, standard deviation.
