# Supplementary material for: Association of Apoptosis-Mediated CD4+ T Lymphopenia With Poor Outcome After Type A Aortic Dissection Surgery
Source: Front Cardiovasc Med. 2021 Nov 12;8:747467. doi: 10.3389/fcvm.2021.747467 (PMC8632808; doi:10.3389/fcvm.2021.747467)
Supplement: Supplementary Table 2 — Baseline characteristics of patients with MAE and No MAE in prospective cohort. [file Table_2.doc]

**Supplemental Table 2. Baseline characteristics of patients with MAE and No MAE in** prospective cohort

| Variables | **MAE**  **（n = 21）** | **No MAE**  **（n = 19）** | ***p*- Value** |
| --- | --- | --- | --- |
|
| Age, years | 52 ± 10 | 49 ± 11 | 0.344 |
| Male | 18 (86) | 14 (74) | 0.580 |
| Smoking | 8 (38) | 6 (32) | 0.666 |
| Hypertension | 17 (81) | 15 (79) | 1.000 |
| Organ malperfusion | 10 (48) | 1 (5) | 0.008 |
| CD4^+^T cells lymphopenia | 17 (81) | 5 (26) | 0.001 |
| Lymphocyte, 10^9^ /L | 0.7 ± 0.2 | 1.4 ± 0.2 | <0.001 |
| Creatinine, mg/dL | 1.1 (0.9, 1.6) | 1.0 (0.7, 1.3) | 0.189 |
| LVEF, % | 67 ± 6 | 70 ± 5 | 0.102 |
| Symptom onset to surgery, h | 31 (26, 56) | 43 (32, 202) | 0.076 |
| CPB, h | 4.5 ± 1.8 | 3.6 ± 1.9 | 0.148 |
| ACCT, h | 2.0 (1.3, 2.6) | 1.3 (0.8, 2.5) | 0.167 |
| HCA ≥ 30 min | 5 (23.8) | 1 (5.3) | 0.128 |
| HCA temperature,℃ | 26(25, 30) | 28 (27, 30) | 0.104 |
| Time of surgery, h | 8.9 ± 2.9 | 8.1 ± 2.9 | 0.347 |

Values are expressed as number (%), mean(SD) or median(IQR).

Depending on the types of data, the Student t test or Mann-Whitney test or Fisher exact test was applied, and p < 0.05 was considered to indicate statistical significance.

ACCT, aortic cross-clamp time; CPB, cardiopulmonary bypass time; HCA, hypothermic circulatory arrest; LVEF, left ventricular ejection fraction; MAE, Major adverse events.
